# Limits in the Perception of Facial Symmetry—A Prospective Study

**DOI:** 10.3390/jpm14111109

**Published:** 2024-11-18

**Authors:** Friederike Lisa Eißing, Dieter Dirksen, Christoph Runte, Susanne Jung

**Affiliations:** 1 Fachklinik Hornheide, Department of Cranio-Maxillofacial Surgery, Dorbaumstraße 300, 48157 Münster, Germany; 2 Department of Prosthetic Dentistry and Biomaterials, University of Münster, Albert-Schweitzer-Campus 1, Building W30, 48149 Münster, Germany; 3 Department of Maxillofacial Surgery, University of Münster, Albert-Schweitzer-Campus 1, Building W30, 48149 Münster, Germany

**Keywords:** facial symmetry, fringe projection technique, asymmetry value

## Abstract

**Objectives:** It is generally accepted that the symmetry of the face plays a significant role in the visual perception of its attractiveness. Therefore, its objective assessment could be useful for individual therapy planning. However, there is an ongoing debate about whether completely symmetrical faces are less attractive than those with minor deviations. The aim of this study is to find thresholds of symmetry perception among faces with an increased spectrum of asymmetry values. **Methods:** The faces of 50 persons (25 men, 25 women) were digitized using a 3D scanner based on the fringe projection technique, and asymmetry values were calculated. In order to achieve a larger spectrum of asymmetry values, some of the surfaces were symmetrized or the symmetry was reduced. Afterward, an independent second group of 50 persons (13 medical professionals, 37 laypersons) rated “symmetry”, “attractiveness” and “health” using a visual analog scale (VAS). **Results:** Symmetry ratings and asymmetry value had a strong and monotonically decreasing association (rho = −0.78, *p* ˂ 0.001). Manipulated or naturally asymmetrical faces (*n* = 12) could not be well distinguished with regard to their symmetry (rho = −0.14, *p* = 0.67). The same applies to very symmetrical or symmetrized faces (*n* = 10, rho = −0.14, *p* = 0.67). Medical professionals rated the symmetry (*p* ˂ 0.001) and attractiveness (*p* ˂ 0.001) significantly higher than laypersons, while there was no significant difference in the health assessment (*p* = 0.24). **Conclusions:** It could be shown that there are indications of threshold values in the perception of facial symmetries, both in the direction of very symmetrical faces and in the direction of asymmetrical faces. There is no evidence that completely symmetrical faces are perceived as less attractive. Thus, in maxillofacial surgery, treatment should aim for the highest symmetry possible, although small deviations are not detrimental.

## 1. Introduction

The face is the focus of attention and plays an important role in perception. Therefore, asymmetries in the area of the face are particularly noticeable and changes in the context of oral and maxillofacial surgery are of considerable importance [1]. Facial symmetry is associated with attractiveness [2,3]. Attractiveness seems to have a “halo effect” to which other people attribute a whole range of positive characteristics and qualities. In our society, good-looking people are considered nicer, more intelligent, more interesting, healthier and more socially competent than their less attractive fellow human beings [3,4]. Regardless of subjective views, an objective assessment of individual facial symmetry can make a useful contribution to a personalized risk–benefit analysis with regard to planned therapy.

In addition to symmetry, averageness, sexual dimorphism and youthfulness are also considered factors that increase attractiveness [3,4]. In addition to the influence of sex chromosomes on the shape of the face, other factors, like Fgf8, located on chromosome 10 (locus 10q24.32), have also been identified to affect the shape of the jaw, for example. Fgf8 mutants may exhibit facial asymmetry with the left side being more affected than the right one [5]. Facial asymmetry is weakly but significantly correlated to dental arch asymmetry; therefore, orthodontic treatment can affect not only the esthetics of the smile but also facial symmetry [6,7]. Asymmetries of the face are already present in early childhood and change during growth. Launonen et al. observed changes in facial asymmetry in children up to the age of 6 years with a measurement method similar to that used in this study. They were able to show that the lower face tends to deviate to the right and the upper face to the left [8]. Generally, a positive correlation of the asymmetry with age has been observed [9]. Facial asymmetry can have different functional and esthetic consequences and subjective perception can also vary. Therefore, an understanding of the perceptual process and the normal range is required [10]. In order to investigate the influence of symmetry on perception, the faces in some studies are symmetrized in different ways. On the one hand, one-sided mirrored faces can be used (chimeric); on the other hand, a face can be symmetrized by morphing. Perrett et al. [11]. reported that the chimeric technique used in some studies to create symmetrical faces leads to structural anomalies in terms of facial proportions and texture [11].

A 2017 review by Wang et al. summarizes the findings on perceptual limits of facial symmetries [12]. Based on the findings from studies, they assumed a sigmoidal relationship between the perceived asymmetry of faces and increasing asymmetry [12]. Previous studies examined individual parts of the face regarding a threshold value. Meyer-Marcotty et al. investigated the perception of the symmetry of three-dimensionally morphed faces with gradual manipulation of the nose and chin along the plane [13]. A deviation of the chin of 6 mm measured from the center could be recognized by most evaluators, whereas a deviation of the nose could be recognized at 4 mm [13]. In a similarly structured study by Dong et al., the observers could clearly perceive chin asymmetries when the deviation reached 4 mm or more [14].

Other studies based on two-dimensional photographs found different thresholds depending on the part of the face examined [15,16,17,18,19,20]. Hohman et al. manipulated a face according to facial expressions (eyebrow elevation, eye closure and smile) in 1 mm increments. In more than 90% of the cases, asymmetries in eye closure were perceived from 2 mm and in smiling from 3 mm onwards [19]. A related study by Chu et al. came to similar results [17]. Asymmetries of at least 3 mm of the oral commissure or the brows, or both, were necessary to be perceived [17]. Ptosis was perceived starting from asymmetries of approx. 1.5 mm [18]. In faces with neutral expression, limits for symmetry perception were found in the area of the nose of 4 mm. Chin deviations of less than 3 mm (rotated around the subnasal point) were not noticed by laypersons [21]. Simulations of unilateral cleft lip were perceived by more than 90% of the subjects from 1 mm asymmetry. Shorter viewing times (3 s vs. 10 s) required a higher degree of asymmetry (2 mm) [16].

So far, studies have been conducted that analyze thresholds for the perception of asymmetries in the face related to specific facial regions [13,14,17,19,20]. The asymmetries were gradually increased and then the evaluation was carried out by another group of subjects.

In our study, the perception of three-dimensionally recorded faces was investigated, for each of which an asymmetry value was determined. In order to achieve a broader distribution of the asymmetry indices of the different faces, this involved symmetrizing some faces and reducing the symmetry of others. So far, to our knowledge, no study has been conducted with such an extended spectrum of faces with different asymmetries. In addition, we analyzed whether there is a difference in symmetry assessment between laypersons and medical professionals.

## 2. Materials and Methods

### 2.1. Participants

In this prospective monocentric study, the facial surfaces of 50 healthy subjects of Caucasian origin were scanned and then presented to a second group consisting of 50 raters. The attributes “symmetry”, “attractiveness” and “health” were assessed using visual analog scales.

The selection of the participants of group 1 (facial stimuli) was carried out randomly from persons from the university environment (University of Münster, Faculty of Medicine). Exclusion criteria in the first group were pathological deformities of the face and, in men, if a beard was worn. In total, 25 women aged 21–35 years (mean: 24.72, SD: 4.06) and 25 men aged 21–31 years (mean: 25.04, SD: 3.43) were recruited.

The participants of group 2 (raters) were recruited on a voluntary basis from the private environment. One prerequisite was that they did not know the subjects from the first group. Therefore, an exclusion criterion was affiliation with the same university institution. They would also have been excluded if they had recognized someone when asked. Further exclusion criteria were known mental disorders or the use of medication that impairs judgment. This group was also composed of 25 women aged 21–62 years (mean: 38.76, SD: 13.49) and 25 men aged 22–74 years (mean: 41.16, SD: 14.33).

All participants in both groups were of Caucasian origin and came from Germany. The participants were informed in detail about the procedure and aim of the study and gave their written consent.

This study was reviewed and approved by the Ethics Committee of the Medical Association of Westphalia-Lippe and the Department of Medicine, University of Münster, Germany, study code 2017-656-f-S.

### 2.2. Optical 3D Recording of the Face Surfaces

**Figure 1:**
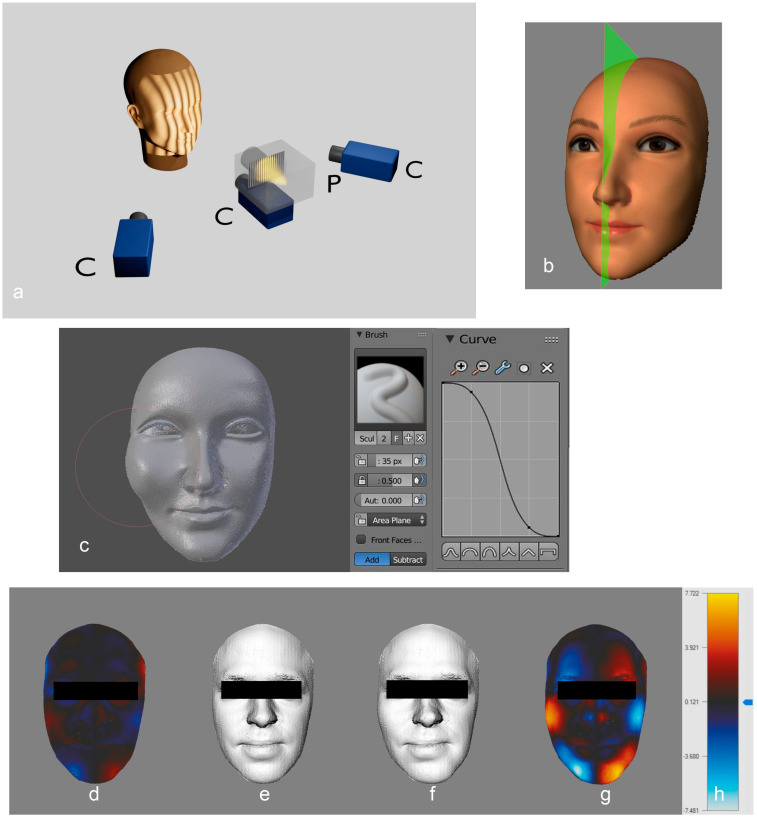
(**a**) Setup of the 3D-scanner system based on fringe projection technique (C = camera; P = projector); (**b**) representation of the plane of symmetry. The 3D data set is limited by the bounding box (pink cuboid). Illustration with the aid of a model. (**c**) Illustration of the user interface in “Sculpt mode” (Blender). For demonstration purposes, an exaggerated deformation of the right cheek is shown. The area inside the pink circle is modified. The size and the extent of the modification can be adjusted (middle). The extent of the deformation follows a sigmoidal course in the surface profile (right; **d**–**h**). Three-dimensional image of a test person with a false color representation of the asymmetry as the difference between the original and mirrored surface (**d**) and discolored (**e**) to demonstrate the asymmetry. After manipulation, the increased asymmetry on the left lower jaw, the right cheekbone and the left forehead (**f**,**g**) are visible. The asymmetry index is 2.38 before the manipulation and 5.9 afterward. The color scale (**h**) representing the distances is shown on the right (scale in mm). The deviation after manipulation is about 7.7 mm at the chin.

The device used is based on the fringe projection technique and has been developed at the University Hospital Münster [22]. Image sequences are generated by three Charge coupled Device (CCD) cameras with a resolution of 1024 × 768 pixels (Imaging Source, Bremen, Germany), which capture the face from different perspectives (Figure 1a). During the recording, a sequence of binary and sinusoidal fringe patterns is projected onto the face using an Liquid Crystal Display (LCD) video projector (NEC VT 58, NEC Corporation, Tokyo, Japan). The surface topography is then reconstructed photogrammetrically, with fringe patterns on the face surface being used to detect corresponding points in the images.

### 2.3. Processing of the Digitized Facial Surfaces

The three-dimensional facial surface was reconstructed as a triangle mesh from the original point clouds consisting of around 150,000 3D coordinates. The presentation and processing of the facial surfaces were carried out with domestic software developed in our department [23]. The acquired data sets were cropped to obtain the facial section (Figure 1b). The points were then re-meshed using Delaunay triangulation.

### 2.4. Processing a Range of Asymmetry Values

In order to obtain an expansion of the spectrum of asymmetry values, some faces were symmetrized (*n* = 10), while, for others, symmetry was reduced (*n* = 12). The symmetrization was conducted by mirroring the point cloud and calculating a weighted mean value for each surface point and its mirrored counterpart. This ensures that both hemispheres contribute equally to the result. The degree of symmetrization was given in %. As a result of the computational operations, a correspondingly symmetrized facial surface was obtained based on the original data set. The reduction in symmetry was carried out with the help of Blender 2.79b (Blender Foundation, Amsterdam, The Netherlands). Employing the sculpt-mode surface, meshes were edited by deforming the triangle mesh with a “brush tool” (Figure 1c). The size of the brush tool and the strength of the deformation can be adjusted. The selection was made randomly based on the asymmetry value. The facial surfaces were processed in such a way that existing asymmetries were enhanced, if possible, to achieve a natural-looking result. The asymmetry index was then recalculated. An example of the manipulations is shown in Figure 1d–h. With the help of the “Edit Mode” in the Blender program, the highlights in the pupil of some faces were added.

### 2.5. Calculation of the Asymmetry Index

The calculation of the asymmetry value was carried out with domestic software, following an approach proposed by Benz et al. [24]. The point cloud was mirrored along the x-y axis as the initially estimated median sagittal plane. Then, the mirrored facial surface was aligned with the original facial surface. For this purpose, the ICP (Iterative Closest Point) algorithm was used to minimize the average distance between the two surfaces by transforming the mirrored surface. A new estimate of the mirror plane was then created by fitting a plane to the centers of gravity of corresponding point pairs of the original surface and the mirrored surface using a least squares method. The original surface was then mirrored and the whole process was iterated until the final plane was regarded as the plane of symmetry. A similar procedure was also used in comparable studies, for example, by Launonen et al. [8]. Finally, an asymmetry index was calculated by dividing the average distance between the point clouds of the original and the “matched” mirrored face surface by the diagonal of its bounding box [23].

### 2.6. Presentation of the Faces in Rotating View and Evaluation by the Rater Group

**Figure 2:**
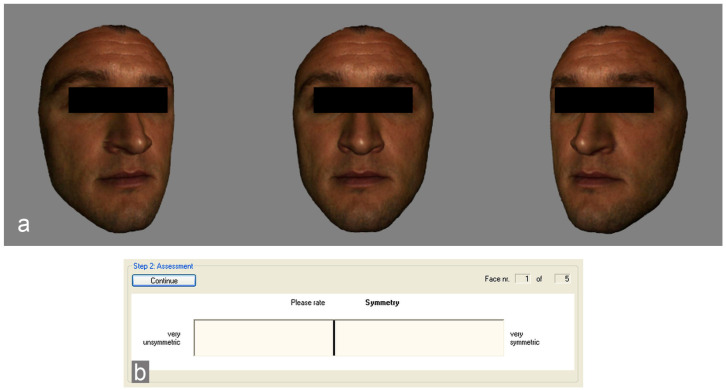
(**a**) The facial surfaces were displayed in a rotation sequence that covered an angular range of 40 degrees. In total, the sequence contained 41 frames and took 4 s; (**b**) image of the digital visual analog scale.

The recorded and manipulated facial surfaces were presented to a second group of test persons (*n* = 50) consisting of 25 women and 25 men in a rotation sequence (Figure 2a). Following the presentation, the subjects were asked to rate the characteristics “symmetry”, “attractiveness” and “health” via a digital visual analog scale, and the results were given in the form of 0 to 100%. The user interface of the Presenter program with the visual analog scale (VAS) is shown in Figure 2b. Here, the order of the evaluation criteria and the order of the presented faces changed randomly. Before each evaluation, the face was presented again. There was no time limit for the evaluation.

### 2.7. Evaluation of the Data

The analysis of the data was carried out with the software “R” (version 4.2.2) for statistical computing [25]. The Shapiro–Wilk test was used to test for normal distribution. The graphs were created with R and the package “ggplot2”. In the present study, most of the asymmetry data were not normally distributed, so the Spearman rank correlation was applied. The rank correlation coefficient according to Spearman’s rho and the *p*-value were determined. The significance level was 5%; 95% confidence intervals were determined for the correlation analyses applying a bootstrapping method (“spearman.ci” function from the “RVAideMemoir” package).

In order to determine the upper and lower thresholds of the measured asymmetry with respect to the rated symmetry, logistic functions were fitted using the package “drc”.

(1) $$f\left(AI\right)=c+\frac{d-c}{1+e^{b(AI-e)}}$$

In this formula, *AI* denotes the asymmetry index. 

Furthermore, a group explorative analysis of the rater groups was carried out. One group consisted of persons who were presumed to have experience in the field of symmetry assessment. This group included oral and maxillofacial surgeons and dentists, who were grouped as “medical professionals”. The other group was called “laypersons”. The paired *t*-test was used for comparison of two related samples. The Wilcoxon signed-rank test was used as an alternative to the *t*-test if normal distribution could not be assumed. 

In order to quantify the error of the rated symmetries, the measured symmetry *SI* was defined as follows:

(2) $$SI=\frac{\max\left(AI\right)-AI}{\max\left(AI\right)-min(AI)}\times 100$$

Each asymmetry value was thus converted into a complementary symmetry value. The symmetry error is then calculated as follows:

*Symmetry error* = *symmetry rating* − *SI* (3)

## 3. Results

### 3.1. Stimuli

**Figure 3:**
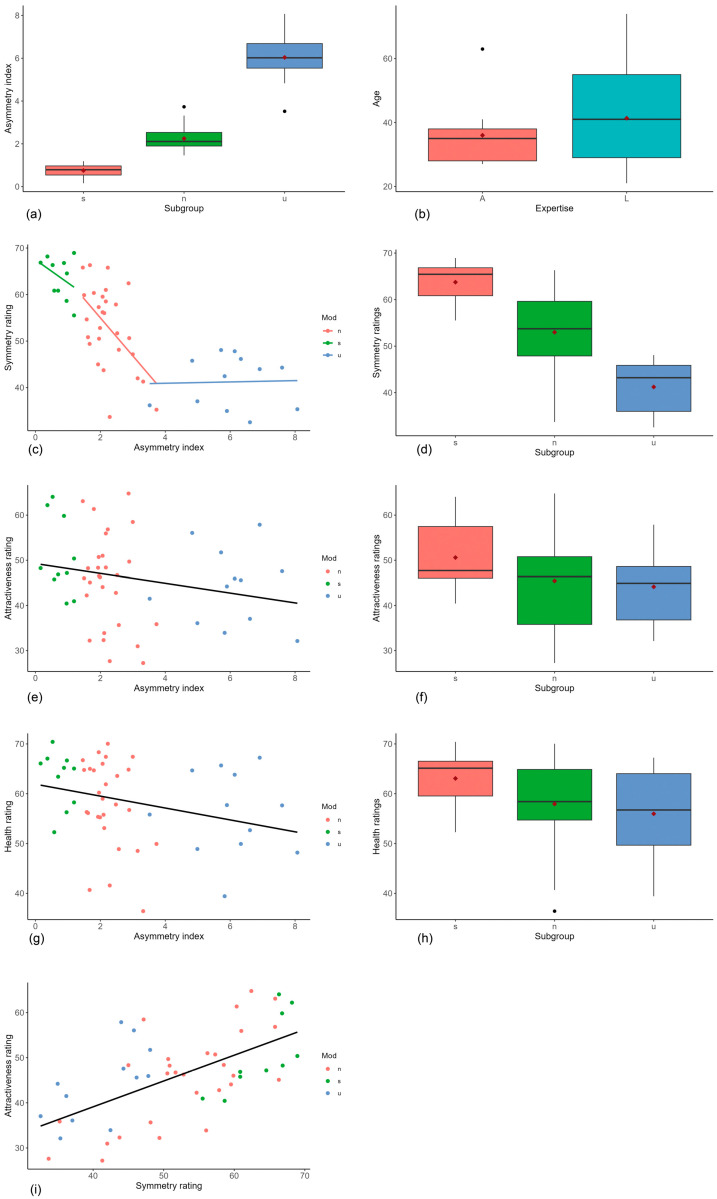
(**a**) The distribution of the asymmetry index of the three subgroups “symmetrized”(s), “not processed” (n) and “reduced symmetry” (u); (**b**) the distribution of the age according to qualification of the participants acting as raters (A = “medical professionals”, L = “laypersons”); (**c**) scatter diagram of the mean symmetry rating per face vs. the asymmetry index with partial regression lines (n = not processed, s = symmetrized, u = reduced symmetry); (**d**) boxplot of the symmetry ratings depending on subgroup (n = not processed, s = symmetrized, u = reduced symmetry); (**e**) scatter plot of the attractiveness rating depending on the asymmetry index (n = not processed, s = symmetrized, u = reduced symmetry); (**f**) boxplot of attractiveness ratings dependent on subgroup (n = not processed, s = symmetrized, u = reduced symmetry); (**g**) scatter plot of health rating depending on symmetry rating (n = not processed, s = symmetrized, u = reduced symmetry); (**h**) boxplot of the health ratings depending on subgroup (n = not processed, s = symmetrized, u = reduced symmetry); (**i**) scatter plot of attractiveness rating depending on symmetry rating (n = not processed, s = symmetrized, u = reduced symmetry). Black dots in the boxplots indicate outliers, red squares mean values.

The distribution of the asymmetry index of the three subgroups “symmetrized” (mean: 0.75, SD: 0.34), “not processed” (mean: 2.25, SD: 0.58) and “reduced symmetry” (mean: 6.04, SD: 1.24) of the presented facial stimuli are shown in Figure 3a. An ANOVA test followed by a Tukey post hoc test revealed significant differences between all groups (*p* < 0.001).

### 3.2. Symmetry Ratings

Figure 3d illustrates a boxplot of the symmetry ratings of the subgroups (n = not processed, s = symmetrized, u = symmetry reduced). The ANOVA test (*p* < 0.001) followed by a Tukey post hoc analysis revealed significant differences between ratings of the subgroups s (mean: 63.75, SD: 4.52), n (mean: 52.99, SD: 8.78) and u (mean: 41.24, SD: 5.6). 

There was a significant correlation between the symmetry rating and the asymmetry index (rho = −0.78, *p* ˂ 0.001). The scatter diagram (Figure 3c) shows the mean symmetry rating per face vs. the asymmetry index of the faces, including the partial dependencies of the subgroups. The analysis shows that the asymmetrical faces (*n* = 12) were rated almost constant in terms of symmetry despite different asymmetry indices (rho = −0.14, CI = (−0.64; 0.41), *p* = 0.67). Symmetrized faces (*n* = 10) were perceived as more symmetrical, but asymmetries could only be differentiated less precisely (rho = −0.32, CI = (−0.95; 0.51), *p* = 0.36). The symmetry score of the unedited faces (*n* = 28) indicates a monotonically decreasing correlation with the asymmetry index (rho = −0.44, CI = (−0.73; −0.018), *p* = 0.02). 

There was no significant correlation (Spearman) between the asymmetry value and attractiveness rating (rho= 0.28, CI = (−0.52; −0.024), *p* = 0.053), whereas a relatively strong association between the symmetry rating and attractiveness rating was found (rho = 0.59, CI = (0.36; 0.74), *p* ˂ 0.001) (Figure 3e,i). The health rating was significantly higher for more symmetrical faces (rho = −0.35, CI = (−0.59; −0.076), *p* = 0.013) (Figure 3g). The corresponding boxplots of the subgroups are illustrated in Figure 3d,f,h.

### 3.3. Evaluation of Groups with Different Expertise

The age distribution of the group of rating persons depending on profession is shown in Figure 3b.

**Figure 4:**
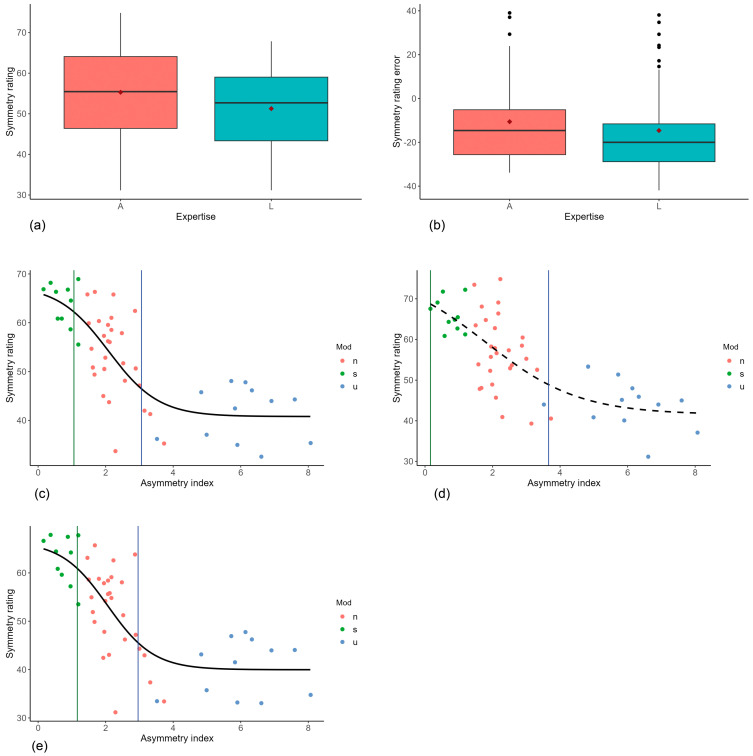
(**a**) Symmetry evaluation according to expertise (A = “medical professionals”, L = laypersons); (**b**) symmetry evaluation error according to expertise (A = “medical professionals”, L = “laypersons”). Black dots in the boxplots indicate outliers, red squares mean values.; (**c**) scatter plot of symmetry ratings vs. asymmetry index with fitted logistic curve (n = not processed, s = symmetrized, u = reduced symmetry). Based on the curvature, the threshold values are an asymmetry index of approx. 1.1 and 3.1; (**d**) scatter plot of symmetry ratings of the medical professionals vs. the asymmetry index with fitted logistic curve (n = not processed, s = symmetrized, u = reduced symmetry). The fit revealed non-significant values for parameters b (*p* = 0.40) and e (*p* = 0.43) in Equation (1). Based on the curvature, the threshold values are at asymmetry index of approx. 0.16 and 3.66; (**e**) scatter plot of symmetry ratings of the laypersons vs. the asymmetry index with fitted logistic curve (n = not processed, s = symmetrized, u = reduced symmetry). Based on the curvature, the threshold values are at AI of approx. 1.16 and 2.96.

Figure 4a shows the symmetry evaluation of the two groups, “medical professionals” and “laypersons”. Overall, the average symmetry rating is significantly higher (*p* ˂ 0.001) in the “medical professionals” group with a mean of 55.5 and a median of 55.46 (minimum 31.15, maximum 74.85), while the mean score for the “laypersons” is 51.27 (median 52.70, minimum 31.16, maximum 67.86).

Figure 4b shows the symmetry evaluation error of the two groups.

According to the Wilcoxon signed-rank test, the symmetry scores of the “medical professionals” and “laypersons” differ significantly (*p* ˂ 0.001). The “medical professionals” have a smaller error and thus scored more accurately (Figure 4b).

### 3.4. Thresholds

Figure 4c shows the fitted logistic function (Equation (1)). The curve runs almost horizontally in the area of asymmetrical faces. Towards the more symmetrical faces, the curve flattens but does not reach a plateau. The shape of the curves could be used to define possible threshold values. According to numerical analysis, the two extreme values of the curvature are at an asymmetry index of approximately 1.1 and an asymmetry index of approximately 3.1.

In Figure 4d,e, the logistic curve has been separately fitted to the symmetry ratings of the medical professionals and the laypersons, respectively. In the case of the medical professionals, the fitted parameters with threshold values of 0.16 and 3.66 were not significant. However, the course of the curve (dotted line in Figure 4d) could be a hint that the interval in which medical professionals are able to differentiate facial symmetry exceeds that of laypersons. The logistic regression for the latter reveals thresholds of 1.16 and 2.96, which are values close to that of the combined groups (Figure 4e).

## 4. Discussion

In our study, it turned out that facial symmetries could be recognized reliably in the middle range. In areas of high symmetry and increasing asymmetry, the ability to differentiate decreased. Threshold values, therefore, seem to exist at the asymmetry index values of 1.1 and 3.1. These findings are consistent with previous findings in the literature review by Wang et al. [12]. Based on various studies, wherein different methods were applied to examine specific facial regions for threshold values, they assumed a sigmoidal progression of the symmetry rating and the calculated asymmetry [12]. In contrast to the studies included in the review, a spectrum of different asymmetries was used and partly extended in our own study. Furthermore, the rating was performed spontaneously based on a rotation sequence, whereas, in the mentioned studies, 2D images were shown.

Previous studies have in common that faces were gradually reduced in symmetry [13,17,19,20,26]. Measurements in the studies were carried out according to the landmark technique, and absolute values for the asymmetry were determined. Threshold values of the individual facial regions vary [13,17,19,20]. Meyer-Marcotty et al. used three-dimensionally manipulated faces that had been gradually modified (2, 4, 6 and 8 mm along the symmetry plane). They were able to show that a deviation of the nose was more noticeable than an asymmetry in the area of the chin [13]. The asymmetries were usually created gradually in only one region of the face, which can lead to jumps. This means that the intermediate steps can be very large. Therefore, an exact differentiation is difficult with large steps.

Manipulations of certain facial regions play a decisive role in perception, as it is already known from studies that central visual areas in particular are observed [27]. In our study, the preferred region for the reinforcement of asymmetries was manipulation of the chin and the cheek, so they would only be recognized at a larger scale. According to a study by Kaipainen et al., most asymmetry is found in the chin and cheek areas and less in the lip, nose and forehead areas [28].

One difficulty in interpreting the results is that the artificially created asymmetries could give an unnatural impression. Further investigations with unprocessed faces should be carried out.

It became apparent that “medical professionals” rated the symmetry significantly higher and more accurately than “laypersons”. There are different results in the literature in this respect. Zamanian et al. found that raters’ profession did not influence the point at which they identified asymmetry [29]. Meanwhile, Dong et al. found a significant difference in the symmetry assessment of deviations of the chin based on three-dimensionally manipulated faces between “dental professionals” and “laypersons” [14]. In this regard, further studies should be conducted with larger and equally sized groups.

There was no significant correlation (in terms of the Spearman correlation) between the asymmetry index and attractiveness rating, whereas a strong association between symmetry rating and attractiveness rating was found. This observation could support the assumption that while symmetry is an important constituent of facial attractiveness, not all types of asymmetries as measured by the asymmetry index contribute equally to this perception. The significance of perfectly symmetrical faces for the perception of attractiveness is the subject of controversial debate. In our study, there is no evidence that completely symmetrical faces are perceived as less attractive. The importance of symmetry for the perception of attractiveness has already been proven in studies [13,30], albeit to varying degrees [31,32]. Surprisingly, some studies in which facial images are processed conclude that a very low degree of fluctuating asymmetry is not associated with maximum perceived attractiveness and that a certain degree of asymmetry is therefore preferred [33,34,35]. In some of the studies mentioned, the technique used to symmetrize faces may have contributed to the symmetrical versions being perceived as less attractive, for example, by mirroring one half of the view or producing artifacts [34,35]. However, a recent study by Harun et al. found that perfectly symmetrical faces generated by image mirroring were rated as more attractive than the original ones [36]. Symmetry is very much a criterion for the positive perception of attractiveness, as some studies, as well as this study, have already shown [13,30]. One limitation of this study is the restriction to a single ethnic group. Future studies should also include other ethnic groups. Another important aspect to be investigated is the dynamic change in facial symmetry during mimic movements, for example.

## 5. Conclusions

It could be shown that there are indications of threshold values in the perception of facial symmetries. Threshold values, therefore, seem to exist at asymmetry index values of 1.1 and 3.1. Perfect symmetry is only perceived to a certain degree. Thus, in maxillofacial surgery, perfect symmetry is not necessary, but it is not a disadvantage either. The thresholds we have determined could be used as a guide in therapy planning.

## Data Availability

The data presented in this study are available on request from the corresponding author due to privacy reasons.
